# Identification and Functional Characterization of a Novel Nonsense Variant in *ARR3* in a Southern Chinese Family With High Myopia

**DOI:** 10.3389/fgene.2021.765503

**Published:** 2021-12-13

**Authors:** Dejian Yuan, Tizhen Yan, Shiqiang Luo, Jun Huang, Jianqiang Tan, Jianping Zhang, Victor Wei Zhang, Yueyuan Lan, Taobo Hu, Jing Guo, Mingwei Huang, Dingyuan Zeng

**Affiliations:** ^1^ Department of Medical Genetics, Liuzhou Municipal Maternity and Child Healthcare Hospital, Liuzhou, China; ^2^ Liuzhou Key Laboratory of Birth Defects Prevention and Control, Liuzhou Municipal Maternity and Child Healthcare Hospital, Liuzhou, China; ^3^ Department of Ophthalmology, Liuzhou Municipal Maternity and Child Healthcare Hospital, Liuzhou, China; ^4^ AmCare Genomics Laboratory, Guangzhou, China; ^5^ Department of Molecular and Human Genetics, Baylor College of Medicine, Houston, TX, United States; ^6^ Center of Breast Diseases, Peking University People’s Hospital, Beijing, China; ^7^ Reproductive Medical Center, The First Affiliated Hospital of Sun Yat-sen University, Guangzhou, China; ^8^ Aegicare (Sheznzhen) Technology Co., Ltd., Shenzhen, China; ^9^ Department of Gynecology, Liuzhou Municipal Maternity and Child Healthcare Hospital, Liuzhou, China

**Keywords:** *ARR3*, NMD, high myopia, cone arrestin, X-linked

## Abstract

*ARR3* has been associated with X-linked, female-limited, high myopia. However, using exome sequencing (ES), we identified the first high myopia case with hemizygous *ARR3*-related mutation in a male patient in a Southern Chinese family. This novel truncated mutation (*ARR3*: c.569C>G, p.S190*) co-segregated with the disease phenotype in affected family members and demonstrated that high myopia caused by *ARR3* is not X-linked, female-limited, where a complicated X-linked inheritance pattern may exist. Thus, our case expanded the variant spectrum in *ARR3* and provided additional information for genetic counseling, prenatal testing, and diagnosis. Moreover, we characterized the nonsense-mediated decay of the *ARR3* mutant mRNA and discussed the possible underlying pathogenic mechanisms.

## Introduction

Myopia occurs when the axial length of the eyes is too long, typically causing distant objects to be focused in the front of the retina (Young, 2009). High myopia (HM) is a common human genetic sensory defect characterized by reduced vision, retinal degeneration, and choroidal atrophy. Its global prevalence exceeds 2.9% (Saw, 2003; Holden et al., 2016). HM is defined by diopters (D) greater than −6.0 or an axial length greater than 26 mm (Young et al., 2007).

Both environmental and genetic factors can influence the severity of myopia (Saw et al., 1996; Morgan et al., 2012), with some family studies showing that genetic factors play a crucial role in the development of HM (Young et al., 2007; Cai et al., 2019). *ARR3* (RefSeq NM_004312.3; see Materials and Methods, “Nomenclature”) gene is associated with X-linked, female-limited HM based on a study of three families where all affected patients were female and hemizygous male family members were asymptomatic (Xiao et al., 2016). Even though several male family members were hemizygous, they did not possess the phenotypic features of HM, thereby proving to be the second disease, after those associated with *PCDH19*, with this unusual inheritance pattern (Depienne et al., 2009).

In this study, a member of a Chinese family was referred for HM, and exome sequencing (ES) revealed that an *ARR3* variant submitted to the LOVD (variant #0000660387) was responsible for the phenotype. Our identification of one male hemizygote with HM symptoms is in stark contrast with the previous report of the female-limited inheritance pattern of *ARR3*-related HM (Xiao et al., 2016), implying that *ARR3* has more complicated hereditary patterns than initially thought.

## Materials and Methods

### Nomenclature

The nomenclature of arrestin protein and gene is very confusing. In fact, the nomenclature of arrestin should be as follows: arrestin-1 (historic names S-antigen, 48 kDa protein, and visual or rod arrestin), arrestin-2 (β-arrestin or β-arrestin-1), arrestin-3 (β-arrestin-2 or hTHY-ARRX), and arrestin-4 (cone or X-arrestin; for unclear reasons, its gene is called “arrestin-3” in the HUGO database) (Zhan et al., 2014). We would like to use *ARR3* (OMIM: 301770) to avoid the confusion in the nomenclature.

### Materials

Plasmid p3XFLAG-CMV-7.1 was a gift from Prof. Zuobin Zhu. The site-directed mutagenesis kit was purchased from Gene Company Limited, Shanghai, China. UPF1 small interfering RNA (UPF1 siRNA) was purchased from RiboBio, Guangdong, China. Monoclonal anti-GFP (B-2) antibody and monoclonal anti-UPF1 antibody were from ABclonal Technology, China. DMEM and fetal bovine serum (FBS) were from Hyclone, USA. DNA polymerase Gold premix was purchased from Tsingke Biotechnology, Beijing, China. The AxyPrepTM PCR Cleanup Kit was from Axygen Biosciences, USA. The TIANprep Mini Plasmid Kit was purchased from Tiangen Technology, Beijing, China. Liposomal Transfection Reagent and the BCA protein quantification kit were purchased from Yisheng Biotechnology, Shanghai, China. RNAiso Plus Total RNA Extraction Reagent, the PrimeScript 1st strand cDNA Synthesis Kit, and the PrimeScript RT Reagent Kit with gDNA Eraser were purchased from Thermo Scientific, Shanghai, China. The SYBR Green Real-Time PCR Master Mix was purchased from Toyobo Biotechnology, Shanghai, China.

### Human Subjects

This study included a family comprising 14 individuals. Of the eight members who were affected, one patient was male. All patients had HM with refractive errors greater than −6.0 D. Participants were recruited at the Division of Medical Genetics at Liuzhou Municipal Maternity and Child Healthcare Hospital, Liuzhou, Guangxi, China. The study was approved by the Ethical Review Board of the Medical Faculty of the Liuzhou Municipal Maternity and Child Healthcare Hospital.

### Exome Sequencing, Variant Calling, and Sanger Sequencing

Exomes sequencing (ES) was performed on the female proband and her parents to identify the causative mutation. The ES protocol achieved a mean coverage of 200x over 98% of the targeted regions. Data were filtered to generate cleanup reads by adapter trimming and reads with base quality < Q20. A minimum coverage of 20x was the standard (AmCare Genomics Laboratory, Guangdong, China) in the regions that contained 5,177 disease candidate genes. The SpeedSeq toolkit was used for the data analysis: 1) performing the human genome alignment with BWA-MEM, 2) marking duplicates using SAMBLASTER, 3) sorting and performing indexing of BAM files with Sambamba, and 4) executing variant calling using FreeBayes. SpeedSeq is an ultra-fast personal genome/exome analysis toolkit for next-generation sequencing (Chiang et al., 2015). It takes roughly 2 h to complete the analysis of one ES sample by using 7 cores 14 threads of the CPU. To quickly and accurately detect pathogenic variants, we used Exomiser to help narrow down the potential candidate variants. Variants were prioritized according to pathogenicity, quality, inheritance pattern, model organism phenotype data, and phenotypes known to be associated with disease genes in humans (Smedley et al., 2015). We removed variants with a minor allele frequency (MAF) < 0.01 in the 1000 Genomes Project, the Genome Aggregation Database (gnomAD), the Exome Aggregation Consortium (ExAC), and the Single Nucleotide Polymorphism Database (dbSNP). We used Exomiser to remove genes that are least to contribute to the phenotype with priorityScoreFilter < 0.501. Sanger sequencing was used to determine the segregation across all family members. The *ARR3* c.569C>G (p.S190*) primers for PCR amplification were 5′-CCA​TAG​GAA​AAC​AGG​CAT​CAG​G-3′ (forward) and 5′-CCT​TGT​TGG​TGC​AGT​TGT​TG-3′ (reverse).

### Plasmid Construction

The full-length *ARR3* cDNA was synthesized and cloned into p3XFLAG-CMV-7.1 to get p3XFLAG-CMV-7.1-ARR3-wt. Subsequently, p3XFLAG-CMV-7.1-ARR3-mut was constructed from p3XFLAG-CMV-7.1-ARR3-wt using a site-directed mutagenesis kit.

### Cell Culture and Transfection

Human retinal pigment epithelial cell line APRE19 was cultured in DMEM, with 10% FBS, 1% penicillin/streptomycin, and 5% of CO_2_ at 37°C. Transfection of p3XFLAG-CMV-7.1-wt/mut was performed with the liposomal transfection reagent according to the manufacturer’s instruction.

### Translation Inhibition

At 40 h after transfection of p3XFLAG-CMV-7.1-wt/mut into APRE19 cells, 20 µg/ml of cycloheximide (CHX) was added to the medium, and translation inhibition was evaluated after 8 h of treatment.

### RNA Interference

p3XFLAG-CMV-7.1-wt/mut was transfected into APRE19 cells, as mentioned above, together with si-RNA UPF1 (50 nmol/ml). Cells were collected for evaluation after 48 h.

### Real-Time Quantitative PCR

For RT-PCR experiments, total RNA was extracted from APRE19 cells using an RNA kit and then reverse-transcribed into cDNAs. The real-time primer pairs upstream of the mutation site were 5′-ACA​AGC​TAG​GGG​ACA​ATG​CC-3 ′and 5′-AAC​CAG​CCG​CAC​ATA​GTC​TC-3′, while the downstream mutation site primers were 5′-ACA​AAG​AGC​TGC​TGG​GGA​TC-3 ′and 5′-CTA​GCG​GCC​TCA​TGA​GAT​GG-3′.

### Statistical Analysis

Three independent experiments were conducted, and the data were presented as mean ± standard deviation (SD). Tukey’s or Dunnett’s test was used for one-way analysis of variance. GraphPad Prism 5 (GraphPad, Inc, San Diego, CA, United States) was used for statistical analysis. *p* < 0.05 was considered to be statistically significant.

## Results

### Clinical Features

**FIGURE 1:**
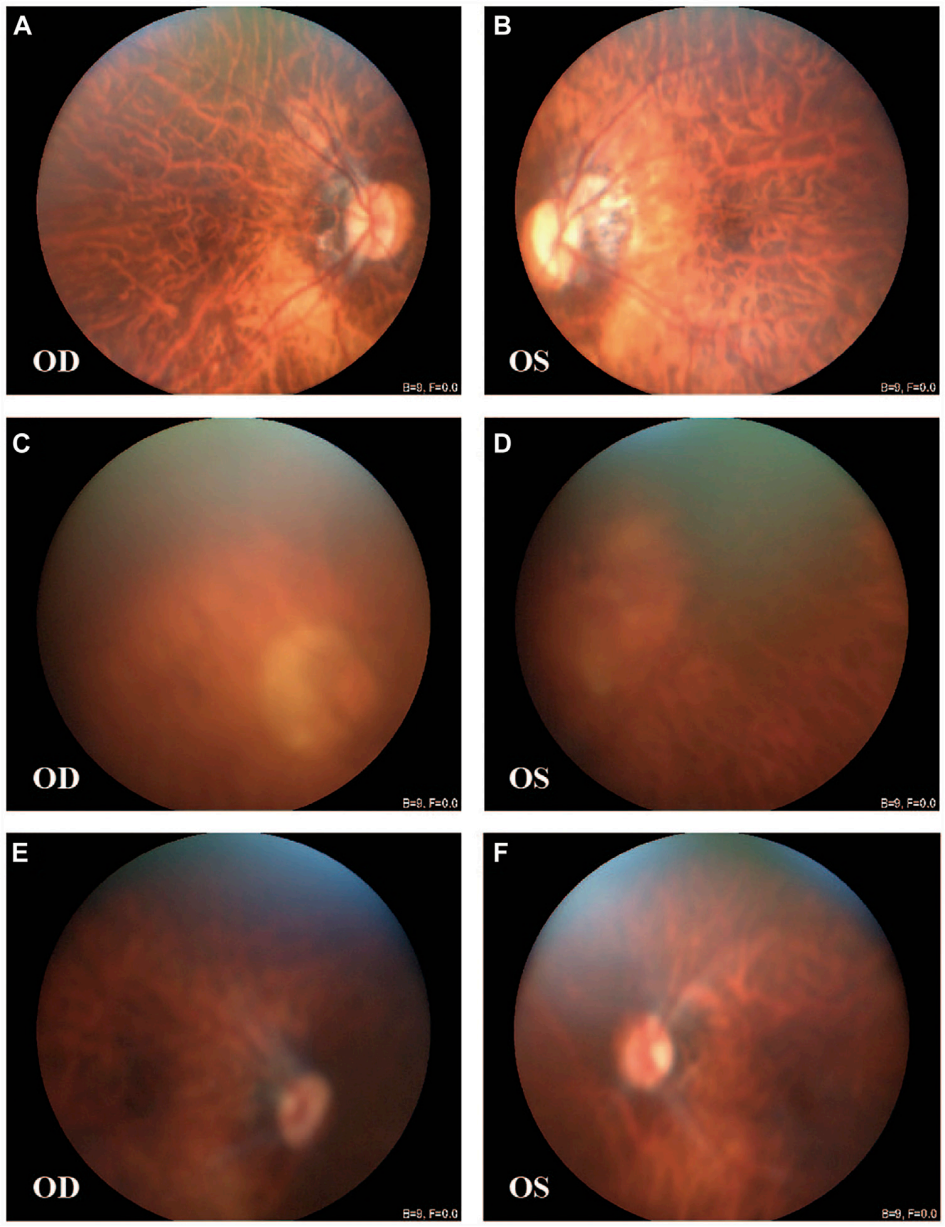
Fundus images of patients from the large family. [III-4: **(A,B)**] Peripapillary atrophy, tigroid appearance, and depigmentation were observed in both eyes. [II-1: **(C,D)**; III-1: **(E,F)**] Tigroid appearance and depigmentation were observed. OD, right eye; OS, left eye.

All patients, including the only affected male family member, had a history of early-onset HM before the age of 7 years, without other ocular diseases or systemic comorbidities. The clinical description of family members is shown in Table 1. Affected members demonstrated peripapillary atrophy, tigroid appearance, and depigmentation changes by examination of fundus photographs (Figure 1). The only male patient in the family (II-1) stated that he had trouble seeing since childhood and began to wear glasses due to myopia when he was 20 years old. His spherical equivalent in the right eye (OD) is −12.00 D and that in the left (OS) is −11.00 D.

**TABLE 1:** Description of the clinical characteristics and the *ARR3* genotypes of the family.

Sample ID	Sex	Age	Affected status	Age at onset	Genotype	Refractive error (D)	
						OD	OS
I:2	F	83	A	NA	c.569C>G/WT	NA	NA
II:1	M	63	A	<7	c.569C>G (HEMI)	−12.00	−11.00
III:1(P)	F	29	A	<5	c.569C>G/WT	−10.00	−11.00
III:2	F	27	A	3	c.569C>G/WT	NA	NA
III:4	F	25	A	3	c.569C>G/WT	−12.00	−13.00
III:6	F	31	A	<5	c.569C>G/WT	−17.00	−17.00
III:7	M	22	U	—	WT	NA	NA
IV:1	F	8	A	3	c.569C>G/WT	−8.00	−7.50
IV:2	F	6	U	—	WT	+0.50	+1.00
IV:3	F	5	A	2	c.569C>G/WT	−7.00	−8.00
IV:4	F	2	U	—	WT	NA	NA

**A**, affected; **D**, diopter; **F**, female; **HEMI**, hemizygote; **M**, male; **NA**, unavailable; **OD**, right eye; **OS**, left eye; **P**, proband; **U**, unaffected; **WT**, wild type.

### Genetic Findings

**FIGURE 2:**
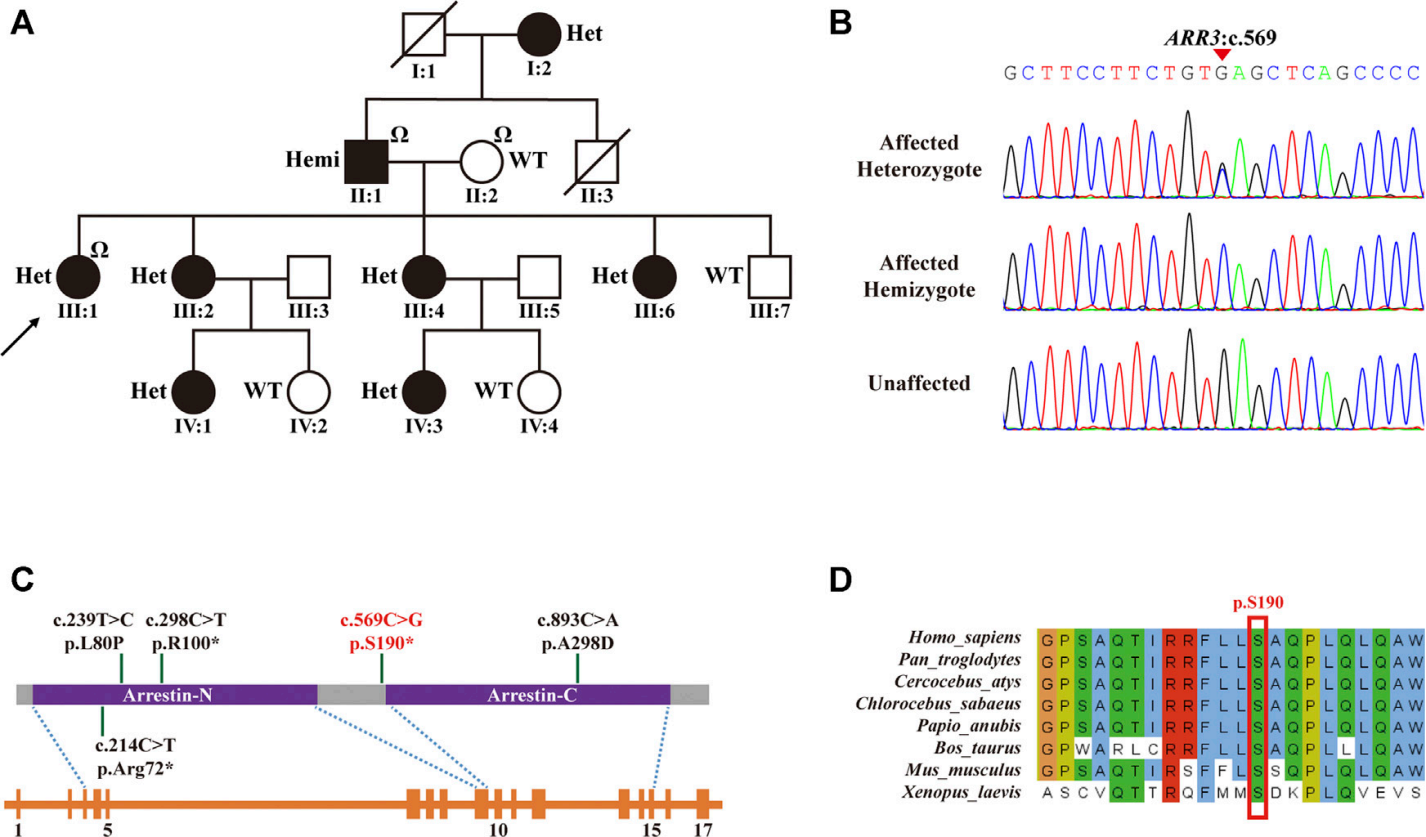
*ARR3* mutation identified in myopia patients. **(A)** Pedigree for the family with the c.569C>G (p.S190*) mutation. Square = male, circle = female, dark symbol = affected, arrow = proband, slash = deceased member, “Ω” = samples used for exome sequencing, Het = heterozygote, Hemi = hemizygote, WT = wild type. **(B)** Sanger sequencing analysis for the mutation identified in the family with high myopia. **(C)** Top to bottom: schematic of the protein structure of ARR3, schematic of the gene structure of *ARR3*, and blocks are exons. The protein contains two important functional domains: an arrestin_N domain and an arrestin_C domain. **(D)** The nonsense *ARR3* c.569C>G (p.S190*) mutation is highly conserved among different species.

Three family members (II-1, father; II-2, mother; and III-1, proband) were selected for ES to identify potential variants responsible for HM in this family. ES analysis revealed a novel heterozygous nonsense mutation in *ARR3* (c.569C>G, p. S190*) in the proband (III-1), which was predicted to produce a shorter, truncated protein product. This mutation was verified by Sanger sequencing and co-segregated with all affected individuals (Figures 2A–D; Table 1). According to ACMG guidelines, the criteria of PM2 (absent from controls) and PP1_strong (greater than 5 affected segregations with HM) were met, and thus, the novel *ARR3* mutation was classified as likely pathogenic.

### Functional Consequences of the Identified *ARR3* Mutation

**FIGURE 3:**
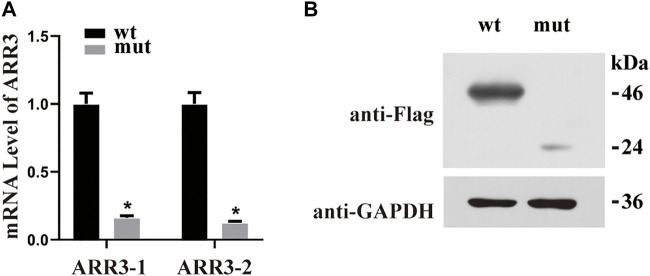
**(A)** Quantitative RT-PCR using *GAPDH* as the housekeeping gene demonstrated that the mRNA expression level was significantly decreased in mutant cases compared with wild-type controls. *p < 0.05 versus WT. **(B)** Western blot analysis of WT and S190* mutant ARR3 in whole-cell lysate.

The human ARR3 protein is composed of 388 amino acids and contains two arrestin-like domains in the N- and C-termini (Figure 2C). To date, there has been only one peer-reviewed report describing three pathogenic *ARR3* variants, and no functional investigations of the underlying molecular mechanisms have been carried out (Xiao et al., 2016). In order to observe the effect of the identified variant on protein expression, the mutant and wild-type proteins were labeled with tag flags, transfected into the APRE19 human retinal pigment epithelial cell line, and cultured for 48 h. The mRNA expression of the mutant gene was significantly lower than that of the wild type (Figure 3A). Furthermore, *ARR3* mutants had significantly lower protein expression than their wild-type counterparts (Figure 3B).

### Degradation of Nonsense *ARR3* Mutant RNA *via* NMD

**FIGURE 4:**
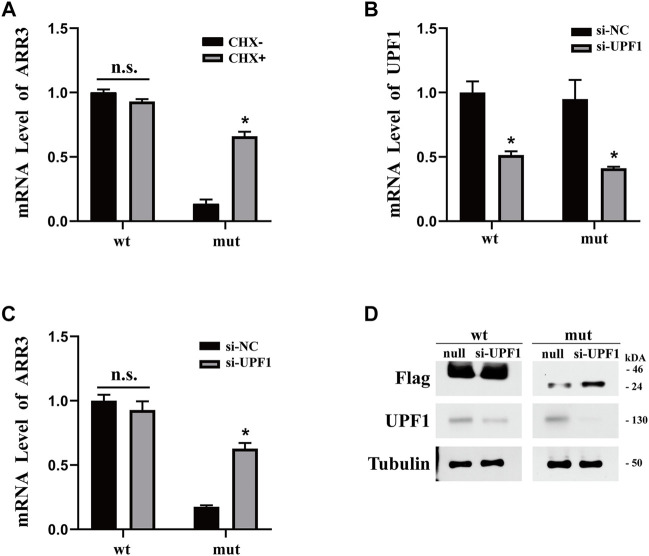
**(A)** CHX treatment increased the mRNA level of mutant *ARR3* but not that of wild-type *ARR3*. **(B)** *UPF1* siRNA treatment significantly decreased the mRNA level of *UPF1*. **(C)** *UPF1* siRNA treatment significantly increased the mRNA level of mutant *ARR3*. **(D)** *UPF1* siRNA treatment significantly increased the protein level of mutant *ARR3* but not that of wild-type *ARR3*.

Nonsense-mediated decay (NMD) has been implicated in the degradation of mRNAs with premature stop codons and may be the reason that we observed a reduced level of mutant *ARR3* mRNA compared to wild type. To test this hypothesis, we first expressed wild-type or mutant *ARR3* in APRE19 cells and then treated the cells with cycloheximide (CHX). CHX is a potent NMD inhibitor, because NMD is translation-dependent and CHX inhibits translation (Noensie and Dietz, 2001). Our results showed that CHX treatment increased the mRNA level of mutant *ARR3* but not that of wild-type *ARR3* (Figure 4A), suggesting that CHX prevents mutant *ARR3* mRNA from degradation through NMD.

To directly test the role of NMD, we knocked down the expression level of *UPF1*, one critical component of the NMD pathway, and then evaluated its effect on the *ARR3* mRNA level. During NMD, UPF1 is the central regulator of UPF proteins to recruit degradation enzymes for the decay of mRNAs with premature termination codons (PTCs) (Kervestin and Jacobson, 2012). As expected, transfection of *UPF1* siRNA leads to efficient downregulation of *UPF1* at both the mRNA level (Figure 4B) and the protein level (Figure 4D). However, knock-down of *UPF1* expression only resulted in a significant increase of the mutant *ARR3* mRNA and protein levels but not those of the wild-type *ARR3* (Figures 4C,D). These data further supported our hypothesis that NMD contributed to the decreased expression of the mutant *ARR3*.

## Discussion

In the present study, we reported an X-linked high myopia case and identified the associated genetic variant in a Southern Chinese family. As reported previously, this nonsense *ARR3* mutation, c.569C>G (p.S190*), co-segregated within affected family members. However, our observation of HM symptoms in a male member was inconsistent with the established female-limited inheritance pattern.

Arrestin is an important family of proteins that can desensitize G-protein–coupled receptors (GPCRs). Rhodopsin belongs to a class of GPCRs that can sense external light signals and transmit these to cells that produce vision. In mammals, there are two types of visual arrestins: arrestin-1 (encoded by *ARR1*, also called S-antigen or 48K protein) (Wacker et al., 1977; Kuhn et al., 1984; Pfister et al., 1985; Vishnivetskiy et al., 2018) and arrestin-4 (encoded by *ARR3*, also called cone arrestin or X-arrestin) (Murakami et al., 1993; Craft et al., 1994). Both arrestins are required for normal inactivation of phototransduction in mouse cones, where it was proposed that arrestin-4 has undergone evolutionary specialization and arrestin-1 maintains the basic function (Nikonov et al., 2008; Deming et al., 2015).

As shown in Figure 2C, of the four previously described pathogenic *ARR3* mutations, two were missense mutations (p.L80P and p. A298D) and two were nonsense mutations (p.R100* and p. R72*), the latter of which would lead to truncated protein translation, similar to our reported c.569C>G (p.S190*). All four mutations could cause functional impairment of arrestin-4 because both the C- and N-terminal domains play a critical role in binding to the receptor molecule (Gurevich and Benovic, 1993; Hirsch et al., 1999). In addition, alignment of multiple arrestin-4 amino acid sequences has shown that the serine at codon 190 is highly conserved (Figure 2D), where evolutionary conservation indicates functional importance.

The pathogenicity of the *ARR3* mutations and their association with HM were described for the first time in 2016, where an X-linked, female-limited pattern of inheritance was proposed based on the observation that none of the hemizygous male family members were diagnosed with HM (Xiao et al., 2016). However, in our study, the proband’s father was affected with HM and possessed this particular pathogenic *ARR3* mutation. This suggested that the hereditary pattern of the *ARR3* gene may not be X-linked, female-limited. HM is a complex, heterogeneous disease which is influenced by environmental and genetic factors. Occurring in females, an X chromosome is compacted during X-chromosome inactivation, which results in the random silencing of one of the X chromosomes (Galupa and Heard, 2018). X-chromosome inactivation could, however, not explain the phenotypes observed in this study since a nonsense mutation was identified in an affected male hemizygote, while unaffected male hemizygotes carried a missense mutation in other families. So we speculate that the mechanism of loss of function caused by null variants may be more detrimental for the *ARR3* gene. Both *Arr1* and *Arr3* genes are expressed in mouse cone cells. If only one of the two genes is knocked out, the recovery time will slow down after being stimulated by saturated strong light, but knockout of both genes will greatly increase the delay in recovery time, which indicates that there may be functional compensation between Arr1 and Arr3 in mice (Nikonov et al., 2008). So it becomes reasonable to question whether there is also a complementary mechanism in the human retina which leads to the occurrence and absence of diseases in different hemizygous individuals? Further proof is needed in the future. *PCDH19*-related epilepsies illustrate an unusual X-linked inheritance pattern (Dibbens et al., 2008). The female heterozygote of the *PCDH19* gene located on the X chromosome is usually patient, but the hemizygous male is usually not affected (Dibbens et al., 2008). Some studies believe that this is a kind of “cellular interference” caused by the cell adhesion affinities of PCDH19 protein (Pederick et al., 2018). Without skewed X chromosome inactivation, the brains of *Pcdh19*+/−female mice showed different cell populations that can adhere improperly to the other. Cells containing mutant Pcdh19 changed the original adhesive condition to another. The presence of two distinctive adhesive properties led to adhesive errors of cells on the developing brain. On the contrary, nerve cells of *Pcdh19*+/+ (or *Pcdh19*−/−) mice sorted suitably since all cells showed the same adhesive condition. Moreover, arrestin appears to have the function of adhesion (Cleghorn et al., 2015). Perhaps there exist a “cellular interference”–like mechanism and other unknown inheritance patterns with incomplete penetrance in *ARR3*, resulting in both normal and affected hemizygous males. This is the goal of our research in the future.

One key limitation of the investigations and discussions mentioned above is the small number of *ARR3*-related HM patients available for further evaluation. Future identification and characterization of more HM-related *ARR3* mutations and their corresponding inheritance patterns will clarify this genotype–phenotype relationship. Only after that can we start to devise plans for precision medicine accordingly.

## Data Availability

The datasets presented in this study can be found in online repositories. The names of the repository/repositories and accession number(s) can be found at: https://db.cngb.org/search/project/CNP0002221/
